# The role of the KIBRA and APOE genes in developing spatial abilities in humans

**DOI:** 10.18699/VJ21.097

**Published:** 2021-12

**Authors:** A.V. Kazantseva, R.F. Enikeeva, Yu.D. Davydova, R.N. Mustafin, Z.R. Takhirova, S.B. Malykh, M.M. Lobaskova, T.N. Tikhomirova, E.K. Khusnutdinova

**Affiliations:** Institute of Biochemistry and Genetics – Subdivision of the Ufa Federal Research Centre of the Russian Academy of Sciences, Ufa, Russia; Ufa State Petroleum Technological University, Department of molecular technologies, Ufa, Russia; Institute of Biochemistry and Genetics – Subdivision of the Ufa Federal Research Centre of the Russian Academy of Sciences, Ufa, Russia; Institute of Biochemistry and Genetics – Subdivision of the Ufa Federal Research Centre of the Russian Academy of Sciences, Ufa, Russia; Bashkir State Medical University, Department of medical genetics and fundamental medicine, Ufa, Russia; Bashkir State University, Department of genetics and fundamental medicine, Ufa, Russia; Psychological Institute of the Russian Academy of Education, Moscow, Russia; Lomonosov Moscow State University, Department of psychology, Russia; Psychological Institute of the Russian Academy of Education, Moscow, Russia; Psychological Institute of the Russian Academy of Education, Moscow, Russia; Lomonosov Moscow State University, Department of psychology, Russia; Institute of Biochemistry and Genetics – Subdivision of the Ufa Federal Research Centre of the Russian Academy of Sciences, Ufa, Russia; Lomonosov Moscow State University, Department of psychology, Russia

**Keywords:** KIBRA, APOE, cognitive abilities, mental rotation, linear regression, gene-environment interactions, KIBRA, APOE, когнитивные способности, мысленное вращение предметов, линейная регрессия, ген-средовые взаимодействия

## Abstract

In the contemporary high-tech society, spatial abilities predict individual life and professional success, especially in the STEM (Science, Technology, Engineering, and Mathematics) disciplines. According to neurobiological hypotheses, individual differences in cognitive abilities may be attributed to the functioning of genes involved in the regulation of neurogenesis and synaptic plasticity. In addition, genome-wide association studies identified rs17070145 located in the KIBRA gene, which was associated with individual differences in episodic memory. Considering a significant role of genetic and environmental components in cognitive functioning, the present study aimed to estimate the main effect of NGF (rs6330), NRXN1 (rs1045881, rs4971648), KIBRA (rs17070145), NRG1 (rs6994992), BDNF (rs6265), GRIN2B (rs3764030), APOE (rs7412, rs429358), and SNAP25 (rs363050) gene polymorphisms and to assess the effect of gene-environment interactions on individual differences in spatial ability in individuals without cognitive decline aged 18–25 years (N = 1011, 80 % women). Spatial abilities were measured using a battery of cognitive tests including the assessment of “3D shape rotation” (mental rotation). Multiple regression analysis, which was carried out in the total sample controlling for sex, ethnicity and the presence of the “risk” APOE ε4 allele, demonstrated the association of the rs17070145 Т-allele in the KIBRA gene with enhanced spatial ability (β = 1.32; pFDR = 0.037) compared to carriers of the rs17070145 CC-genotype. The analysis of gene-environment interactions revealed that nicotine smoking (β = 3.74; p = 0.010) and urban/rural residency in childhood (β = –6.94; p = 0.0002) modulated the association of KIBRA rs17070145 and АРОЕ (rs7412, rs429358) gene variants with individual differences in mental rotation, respectively. The data obtained confirm the effect of the KIBRA rs17070145 Т-allele on improved cognitive functioning and for the first time evidence the association of the mentioned genetic variant with spatial abilities in humans. A “protective” effect of the APOE ε2 allele on enhanced cognitive functioning is observed only under certain conditions related to childhood rearing.

## Abstract (ru)

В современном высокотехнологичном обществе пространственные способности являются предиктором успешности в жизни и профессиональной деятельности, особенно в STEM дисциплинах (от англ. Science, Technology, Engineering, and Mathematics). Согласно нейробиологическим гипотезам, существование индивидуальных различий в когнитивных способностях может быть обусловлено особенностями функционирования генов, участвующих в регуляции нейрогенеза и синаптической пластичности. С другой стороны, полногеномный анализ ассоциаций идентифицировал rs17070145 в гене KIBRA, ассоциированный с индивиду- альными различиями в эпизодической памяти. Учитывая важную роль генетической и средовой компоненты в манифестации когнитивных функций, целью настоящего исследования являлись оценка основного эффекта полиморфных вариантов генов NGF (rs6330), NRXN1 (rs1045881, rs4971648), KIBRA (rs17070145), NRG1 (rs6994992), BDNF (rs6265), GRIN2B (rs3764030), APOE (rs7412, rs429358), SNAP25 (rs363050) и оценка ген-средовых взаимодей- ствий в формировании индивидуальных особенностей пространственного мышления у индивидов без ког- нитивных нарушений 18–25 лет (N = 1011, 80 % женщин). Измерение уровня пространственных способностей осуществлялось с помощью батареи тестовых заданий на вращение 3D фигур (shape rotation). Множественный линейный регрессионный анализ, проведенный в общей выборке с включением половой, этнической принад- лежности и наличия «рискового» аллеля АРОЕ ε4 в качестве ковариат, продемонстрировал ассоциацию аллеля rs17070145*Т в гене KIBRA с более высоким уровнем пространственного мышления (β = 1.32; pFDR = 0.037) по сравнению с носителями генотипа rs17070145*СС. Анализ ген-средовых взаимодействий выявил, что табако- курение (β = 3.74; р = 0.010) и место воспитания в детстве (β = –6.94; p = 0.0002) модулируют ассоциацию по- лиморфных вариантов в гене KIBRA (rs17070145) и гене АРОЕ (rs7412, rs429358) с индивидуальными различиями в пространственных способностях соответственно. Полученные результаты подтверждают связь аллеля rs17070145*Т в гене KIBRA с улучшением когнитивных функций и впервые свидетельствуют об ассоциации дан- ного генетического варианта с особенностями пространственного мышления. «Протективный» эффект аллеля APOE ε2 на улучшение когнитивного функционирования наблюдается только при сочетании определенных особенностей воспитания в детстве.

## Introduction

The study of the productivity of cognitive functions as an integral part of individual potential is becoming increasingly relevant today since the level of cognitive functioning is the basis of individual life success and self-actualization. In particular, in a modern high-tech society, spatial abilities (i. e., ability for 3D mental rotation) predict success in life and professional activity, especially in STEM (Science, Technology, Engineering, and Mathematics) disciplines (Nagy-Kondor, 2017). The hypotheses of an individual trajectory of spatial ability existing to date suggest a significant role of genetic, epigenetic and environmental factors (Mustafin et al., 2020; Takhirova et al., 2021). According to twin research, the impact of the genetic component on individual variance in this trait varies within 64–84 %, depending on the type of examined spatial ability (Malanchini et al., 2020).

According to neurobiological hypotheses, individual differences in cognitive abilities may be due to the specificity of gene functioning involved in the regulation of neurogenesis and synaptic plasticity in such brain regions as prefrontal cortex and hippocampus (Mustafin et al., 2020). The latter process represents the development of neuronal connections as a response to novel experiences. An important role in the regulation of this process belongs to neurotrophic factors (BDNF, NGF ), neurexins (NRXN1), neuregulin (NRG1), synaptosomal-associated protein (SNAP25), glutamatergic receptor (GRIN2B) (Enikeeva et al., 2017; Mustafin et al., 2020). One of the most significant and replicating results obtained in the studies of cognitive functioning is the association of the АРОЕ ε4 allelic variant with an increased risk of developing Alzheimer’s disease and higher rate of cognitive decline (Porter et al., 2018; Li X. et al., 2019). Previous attempts were made to evaluate the effect of different variants of the genes involved in neurogenesis (APOE, TOMM40, BDNF, SORL1, and CLSTN2) on cognitive changes in nondemented individuals above 65–70 years (Laukka et al., 2020). Considering that about 60 % of variance in age-related cognitive changes correlates with different cognitive domains (episodic and semantic memory, information processing speed, nonverbal intelligence, spatial ability, etc.) (Tucker-Drob et al., 2019), it can be assumed that allelic variants of genes, which encode neurogenesis-involved proteins, can also attribute to differences in spatial abilities.

Together with the candidate gene approach, a significant contribution in the study of complex traits is related to such methodological approach as genome-wide association analysis (GWAS), which made it possible to identify genetic variants involved in the regulation of cognitive functioning. The rs17070145 located in intron 9 of the KIBRA (KIdney and BRAin expressed protein) gene, which was initially identified in the GWAS of episodic memory in cohorts from Sweden and America (Papassotiropoulos et al., 2006), represents one of the loci associated with cognitive functioning. Subsequent studies confirmed the association of the minor T-allele with improved episodic memory (Porter et al., 2018) and spatial learning (Schuck et al., 2013). A recent meta-analysis based on 20 case-control studies confirmed the association of the rs17070145 C-allele with an increased risk of developing Alzheimer’s disease and cognitive decline among aged individuals (Ling et al., 2018). It is known that the KIBRA gene (also known as WWC1, WW domain-containing protein 1) encodes a signal transduction protein, which is widely expressed in the kidney and brain regions related to memory regulation (hippocampus, prefrontal cortex, cerebellum and hypothalamus). It is involved in multiple cellular functions, including cell migration, vesicular transport, transcription, synaptogenesis, neuronal signaling, and has a neuroprotective effect, thus inhibiting Aβ-induced apoptosis (Heitz et al., 2016). Moreover, from a functional point of view, reduced Kibra level was shown to mediate memory and synaptic plasticity decrease (Heitz et al., 2016). It should be noted that cognitive functioning can be mediated by an additive and epistatic interaction of proteins encoded by the APOE and KIBRA genes (Wang et al., 2019), which indicates the requirement of simultaneous analysis of both genes.

To date, it remains unknown whether rs17070145 in the KIBRA gene is involved in the regulation of other cognitive abilities (including spatial ability) in individuals of younger age. Therefore, considering that the T-allele in the KIBRA gene was associated with improved memory and executive functions in the majority of studies in individuals without cognitive deficit and was related to better functioning of prefrontal cortex and hippocampus (Papassotiropoulos et al., 2006; Zhang et al., 2009), we suggest that a similar relationship may be observed with enhanced spatial ability in mentally healthy individuals.

Together with a genetic component, individual variance in spatial abilities may be attributed to a specific micro- and macro-environment in ontogenetic development, including sex (Lauer et al., 2019). In this regard, the present study aimed to: (1) estimate the main effect of polymorphic variants of genes involved in neurogenesis and synaptic plasticity; (2) estimate the effect of gene-by-environment interactions on individual differences in spatial ability in individuals without cognitive impairments.

## Materials and methods

The study sample included 1011 mentally healthy young adults (80 % women) – students at Universities of the Republic of Bashkortostan and the Udmurt Republic (mean age 19.79 ± 1.69 years), consisted of Russians – 535, Tatars – 231, Udmurts – 160, and individuals of mixed ethnicity – 85. All volunteers had no individual and familial history of psyhopathologies

The assessment of spatial abilities was conducted in 2017– 2019 via the battery of cognitive tests, which estimated the number of correct answers on the items related to “shape rotation” and were implemented online in the psychodiagnostic platform designed by the Russian Academy of Education. All enrolled individuals filled the inventory consisting of questions on ethnicity by three generations together with several social parameters such as the specificity of child-parent relationship (a style of parental rearing, maltreatment in childhood, rearing in a full/incomplete family), family income level, maternal and paternal age at individual’s birth, place of residence in childhood, sibship size and birth order, bilingual rearing, the presence of chronic disorders and smoking. Place of residence in childhood (urban/rural residency) was determined on the basis of its population size according to (Kazantseva et al., 2020a): demographic locations with population size under 50,000 individuals were determined as a rural residency. All the volunteers filled informed consents for the participation in the study. The study was approved by the Bioethical Committee at the Institute of Biochemistry and Genetics UFRC RAS (Ufa, Russia).

Collection of biological material was performed within 2017–2019 followed by DNA isolation from the peripheral tissue leukocytes. Genotyping of 10 SNPs in the NGF (rs6330), NRXN1 (rs1045881, rs4971648), KIBRA (rs17070145), NRG1 (rs6994992), BDNF (rs6265), GRIN2B (rs3764030), APOE (rs7412, rs429358), and SNAP25 (rs363050) genes was carried out via real-time PCR using KASP kits (LGC Genomics, UK) with CFX96 DNA Analyzer (BioRad, USA) and end-point fluorescence analysis. The АРОЕ genotypes were grouped based on the presence of ε2, ε3, ε4 allelic variants.

Quantitative data were tested for the correspondence to the Gaussian distribution via Shapiro–Wilk W-test ( р > 0.05). The main effect assessment was performed via multiple linear regression analysis. Various statistically significant models (additive, dominant, recessive) were analyzed with PLINK v.1.09, while sex, ethnicity, presence/absence of APOE ε4 allele were included as independent variables (covariates) together with the genotypes (formula (1)). In addition, to analyze gene- byenvironment interactions examined socio-demographic parameters and genotypes were included in linear regression models as independent variables according to formula (2):

**Formula 1.**
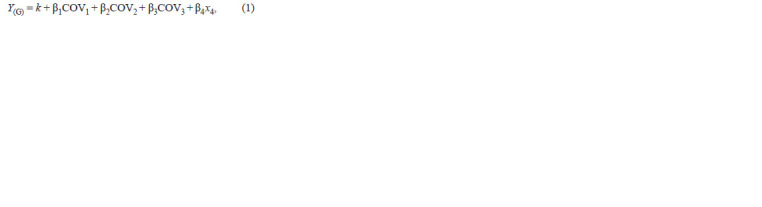
Formula 1.

**Formula 2.**
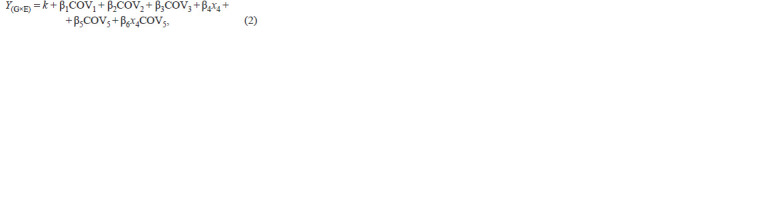
Formula 2.

where Y – spatial ability score; k – intercept; β1, …, 6 – regression coefficients; COV1 – sex; COV2 – ethnicity; COV3 – presence/absence of АРОЕ ε4 variant; x4 – the presence of minor allele of examined SNP in dominant model (the number of copies of minor allele for the additive model); COV5 – environmental predictor; x4COV5 – allele-by-environment interaction.

For statistically significant gene-by-environment interaction model, a stratification analysis between the groups split by either environmental predictor or genetic component was conducted (SPSS 23.0). Correction for multiple comparisons was carried out via FDR procedure (PLINK v.1.09).

## Results

In the present study, allele and genotype frequencies distribution corresponded to the Hardy–Weinberg equilibrium (see the Table). Subsequent multiple regression analysis, which was performed in the total sample controlling for sex, ethnicity and the presence of “risky” АРОЕ ε4 allele, demonstrated the association of KIBRA rs17070145 T-allele with enhanced spatial ability (β = 1.32; βST = 0.10; р = 0.003; рFDR = 0.037; r2 = 0.007) compared to carriers of rs17070145 СС-genotype (in additive model, see the Table).

**Table:**
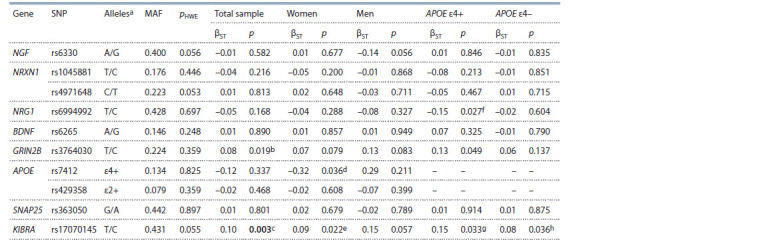
Examined SNPs, the Hardy–Weinberg equilibrium test and the results of linear regression analysis of SNPs association with spatial ability (under additive model) in the total sample and in subgroups Notе. MAF – minor allele frequency; pHWE – p-value for the Hardy–Weinberg test; βST – standardized regression coeff icient; p – p-value for the Wald test. Statistically signif icant differences (after FDR-correction) are shown in bold. a minor/major alleles; b pFDR = 0.098; c pFDR = 0.037; d pFDR = 0.183; e pFDR = 0.183; f pFDR = 0.164; g pFDR = 0.164; h pFDR = 0.368.

**Fig. 1.**
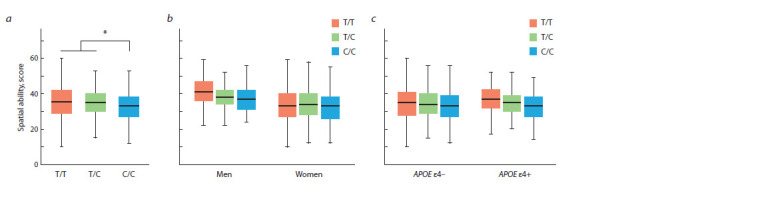
Mean spatial ability scores depending on the KIBRA rs17070145 genotype in the total sample (a), in men, women (b) and the carriers/non-carriers of APOE ε4 variant (c). Statistically significant differences in the mean values of spatial ability between the groups are marked with brackets, *pFDR < 0.05.

Liner regression models, which were applied separately to men, women, АРОЕ ε4 allele carriers/non-carriers, failed to observe a statistically significant effect of the examined loci after correction for multiple comparisons (рFDR > 0.05, see the Table). Mean spatial ability scores depending on the KIBRA rs17070145 genotype in the total sample, as well as in men, women, АРОЕ ε4 allele carriers/non-carriers are shown in Fig. 1.

**Fig. 2.**
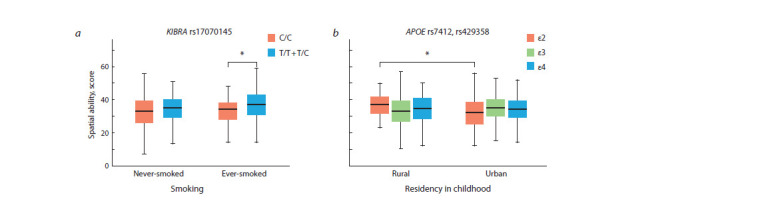
The results of gene-by-environment interaction analysis, which demonstrated a modulating effect of (a) smoking on association of KIBRA rs17070145 variants on spatial ability; (b) place of residence in childhood on association of АРОЕ variants with spatial ability. Statistically significant differences in the mean values of spatial ability between the groups are marked with brackets, * рFDR < 0.05.

As a result of gene-by-environment interactions analysis, which considered both the effect of genetic variants and various social parameters, smoking was revealed to modulate association of KIBRA rs17070145 with individual differences in spatial ability (β = 3.74; βST = 0.14; p = 0.010). To clarify the effect of smoking on cognitive abilities we conducted stratification analysis, which demonstrated that enhanced spatial ability was characteristic for ever-smoking rs17070145 T- allele carriers compared to non-smoking individuals (β = 4.59; βST = 0.22; r2 = 0.003; pFDR = 0.004) (Fig. 2, a). Moreover, the model with inclusion of АРОЕ variants and place of residence in childhood was also statistically significant (β = –6.94; βST = –0.23; p = 0.0002). In addition, higher spatial ability was demonstrated in the carriers of “favorable” APOE ε2 allele, who indicated their rural residency, compared to those from the urban regions (β = –6.04; βST = –0.25; r2 = 0.06; pFDR = 0.021) (see Fig. 2, b).

## Discussion

Since previous research indicated the necessity to control for the well-known “risk” factor of developing cognitive deficit (APOE ε4 variant) in the statistical models (Porter et al., 2018; Li X. et al., 2019), the hypothesis suggested in the present study was examined in both total sample and in the groups split by the presence of APOE ε4 allele. Previous findings evidence that individuals without cognitive decline carrying APOE ε4 variant (related to the accumulation of amyloid beta (Aβ)) demonstrated an increased decline in the verbal episodic memory and hippocampal hypotrophy in the presence of KIBRA rs17070145 СС-genotype compared to minor T-allele carriers (Porter et al., 2018). In the present study, the analysis of individuals aged 18–25 years without cognitive impairments failed to detect significant effect of APOE ε4 allele on the association of KIBRA gene variants with the level of spatial abilities. Nevertheless, we included the mentioned “risk” allele in the APOE gene as an independent variable in multiple regression models. As a result of these analyses, for the first time we demonstrated a positive effect of KIBRA rs17070145 Т-allele on higher spatial ability in individuals without cognitive deficit, which at some extent is congruent to findings obtained by other research groups in non-demented healthy individuals (Schuck et al., 2013; Porter et al., 2018).

The effect observed is confirmed by functional studies, which reported that rs17070145 in the KIBRA gene was related to grey matter volume in the prefrontal cortex and parahippocampal gyrus in elderly individuals (Li R. et al., 2020). In particular, as a result of voxel-oriented morphometry grey matter volume was diminished in carriers of rs17070145 С-allele compared to individuals with rs17070145 TT-genotype in both elderly participants (Li R. et al., 2020), and youngerage volunteers (Wang et al., 2013), which, in turn, reflects an improved cognitive functioning in carriers of minor T-allele. Interestingly, young-age individuals with C-allele, which is associated with reduced grey matter volume, demonstrated a compensatory effect via enhanced synchronization between the brain regions involved in the regulation of executive control (Wang et al., 2013). The results obtained by our group can be explained by an increased level of long-term potentiation (LTP) in hippocampus and associated enlarged cognitive functioning related to an increased expression of the KIBRA gene (Heitz et al., 2016), which can be due to the presence of rs17070145 Т-allele. From the other side, rs17070145 may be in a linkage disequilibrium with other functional variants (resulting in missence mutations such as rs3822660G/T or M734I, rs3822659T/G or S735A), which are located in the exon 15 of the KIBRA gene and mediate differential Ca2+-dependent binding of protein С2-domain with phosphatidylinositol, therefore regulating cellular pathways (Duning et al., 2013).

The researchers suggest that controversy of the impact of KIBRA rs17070145 in published findings may be related to the cognitive status of the examined sample, as well as to demographic parameters including age (Zhang et al., 2009; Li X. et al., 2019). Therefore, we analyzed different regression models, which consisted of various environmental predictors. One of the interesting findings of the present study is the effect of smoking, which was shown to modulate the association of allelic variant in the KIBRA gene with spatial ability. Another research group succeeded to identify that reduced number of constant mistakes in cognitive tests was observed in smoking individuals from European populations compared to neversmoking individuals, but this association was prominent only in carriers of rs17070145 T-allele in the KIBRA gene (Zhang et al., 2009). Notably, in the present study the inclusion of interaction terms (KIBRA rs17070145 genotype and smoking) in the linear regression model also revealed the association of minor T-allele with higher shape rotation ability, which was characteristic for ever-smoking individuals compared to never-smoking ones. Therefore, it was suggested that nicotine might positively affect cognitive abilities (including executive functions and attention) in individuals with T-allele (Zhang et al., 2009). According to our previous research, nicotine may have a modulating effect on genetic association with individual cognitive and psychological domains in mentally healthy individuals (Davydova et al., 2020), which may be caused by nicotine-related changes in epigenetic profile in the examined genes.

Although multiple studies evidence the association of APOE “risky” ε4 allele with cognitive decline and Alzheimer’s disease, diminished grey matter volume in hippocampus (Porter et al., 2018; Li X. et al., 2019), and lower spatial abilities (Laczó et al., 2020), we failed to identify the main effect of APOE gene variants on individual variance in spatial ability in mentally healthy individuals without cognitive decline. Previously, the attempts to estimate a combined effect of APOE ε4 allele and environmental predictors (smoking, physical activity, overweight, education level) on cognitive domains in individuals aged 40–79 years have been performed, which failed to detect statistically significant models of gene-by-environment interactions (Rodriguez et al., 2018). Nevertheless, the analysis of gene-by-environment interactions conducted by our group made it possible to observe the involvement of APOE gene variants in mental rotation ability depending on the place of residence in childhood (rural/urban). The highest spatial ability level was characteristic for individuals bearing “favorable” APOE ε2 allele, who indicated rural residency, compared to urban-residency participants. Accordingly, based on the data obtained it can be assumed that unfavorable effect of urban residency in childhood is even observed in the case of presence of “favorable” APOE ε2 allele related to enhanced neuronal activity (Davis et al., 2020).

Published data from other research groups also indicated a correlation between absent cognitive decline in elderly individuals and the presence of “green” territorial neighborhood in childhood; moreover, this effect was characteristic for individuals without APOE ε4 allele (Cherrie et al., 2018). Interestingly, the presence of available “green” neighborhood positively affected memory and attention in school-aged children even during one year (Dadvand et al., 2015), while long-lasting accommodation in the “green” neighborhood correlated with an enhanced grey matter volume in the prefrontal cortex, thus, explaining improved cognitive functioning (Dadvand et al., 2018). This observation can be explained by several items. First, urban residency is related to higher level of ecopollutants and xenobiotics, which results in impaired regulation of various neurotransmitter systems in the brain (Dadvand et al., 2018). Second, urban/rural residency results in the differences in the lateralization of functions, specificity of language development and visual-spatial processes (Polyakov, 2008). In particular, rural residency is characterized by forced development of right-hemispheric brain structures, whereas urban school-aged children demonstrated predominant development of left-hemispheric functional systems, which reflects the specificity of their cognitive ability. Third, a positive effect of rural rearing on cognitive domains may be related to the nutrition specificity, although only in the absence of “unfavorable” APOE ε4 allele, which was published previously (Martínez-Lapiscina et al., 2014). From the one side, the consumption of eco-friendly available farmer nutrition products by children of rural residency and higher level of their physical activity (including their help to older family members in the gardens) on the other hand may provide the “manifestation” of a positive effect of APOE ε2 allele on spatial ability. Fourth, as a consequence of rural residency, children may obtain a “favorable” gut microbiota content and diversity, which directly affects brain development via gut-brain axis based on the recent research findings (Mancabelli et al., 2017).

On the other hand, population urbanization is accompanied by development of mental and cognitive impairments due to a diminished exposure to macro- and microorganisms, thus resulting in disturbed immunoregulation. In turn, it may result in increased inflammatory response of the body on psychological stressors related to residency in a high-tech society compared to small territorial units (Rook et al., 2013). In addition, the impact of place of residency on cognitive functioning may be attributed to enhanced stress level characteristic for urban residency and correlating with cortisol level. One of the studies demonstrated the interaction between cortisol level and the presence of “risky” APOE ε4 allele, which caused a decline in cognitive functioning (Lee et al., 2008).

Therefore, the demonstrated effect of gene-by-environment interactions, which is related to the spatial ability development, observed in the present study allows us to suggest that a positive effect of the APOE ε2 allele on cognitive abilities may be identified only under rural residency, which is presumably related to the favorable impact on neuronal processes.

Despite the possible epistatic or additive effect of interactions of the genes involved in neurogenesis and synaptic plasticity (Wang et al., 2019), we failed to observe either the main or epistatic effect of the BDNF, NGF, NRXN1, NRG1, SNAP25, and GRIN2B genes on interindividual differences in spatial ability. Other studies also revealed no association of neurogenesis-related gene variants (APOE, SORL1, BDNF, TOMM40, KIBRA, and COMT ) with spatial abilities in individuals aged 40–60 years (Korthauer et al., 2018). According to our previous research, several genes involved in synaptic plasticity regulation such as the SNAP25, NRXN1, and NRG1 are responsible for individual differences in such cognitive domains as mathematical abilities (Kazantseva et al., 2020b) and working memory volume (Enikeeva et al., 2017). Despite the suggestion proposed in the present study on the association of allelic variants in neurogenesis-involved genes in spatial ability, we failed to confirm such association.

## Conclusion

As a result of the present cross-sectional study, the main effect of the KIBRA gene on the development of the spatial ability was observed in individuals without cognitive deficit; moreover, respondents’ smoking positively affected the examined cognitive domain in carriers of rs17070145 minor T-allele. It should be noted that we confirmed a “protective” effect of APOE ε2 allele on improved cognitive functioning, which manifested only in the presence of such favorable factor as rural residency in childhood. The data obtained are congruent with previously claimed suggestions on the association of rs17070145 minor T-allele in the KIBRA gene with improved cognitive functioning and primarily evidence the involvement of this genetic variant in individual differences in spatial ability.

The present study has several advantages including a large sample size of similar-age individuals, control for sex, ethnicity and “risky” allele in the APOE gene in regression models (i. e. inclusion of mentioned predictors in multiple linear regression models). The examined sample was collected prior to COVID-19 pandemic, which allowed us to avoid the possible effect of SARS-CoV-2 on nervous system and cognitive functions, which has been repeatedly demonstrated (Fotuhi et al., 2020).

## Conflict of interest

The authors declare no conflict of interest.
